# Gallic acid inhibits neuroinflammation and reduces neonatal hypoxic-ischemic brain damages

**DOI:** 10.3389/fped.2022.973256

**Published:** 2022-12-22

**Authors:** Xiangjun Dong, Shuyue Luo, Dongjie Hu, Ruixue Cao, Qunxian Wang, Zijun Meng, Zijuan Feng, Weihui Zhou, Weihong Song

**Affiliations:** ^1^ Chongqing Key Laboratory of Translational Medical Research in Cognitive Development and Learning and Memory Disorders, Ministry of Education Key Laboratory of Child Development and Disorders, National Clinical Research Center for Child Health and Disorders, China International Science and Technology Cooperation Base of Child Development and Critical Disorders, Children's Hospital of Chongqing Medical University, Chongqing, China; ^2^ Institute of Aging, Key Laboratory of Alzheimer's Disease of Zhejiang Province, Zhejiang Provincial Clinical Research Center for Mental Disorders, School of Mental Health and Kangning Hospital, The Second Affiliated Hospital and Yuying Children's Hospital, Wenzhou Medical University, Wenzhou, China; ^3^ Oujiang Laboratory (Zhejiang Lab for Regenerative Medicine, Vision and Brain Health), Wenzhou, China

**Keywords:** hypoxic-ischemic brain damage, gallic acid, neuroprotective effect, neuroinflammation, neuronal loss

## Abstract

Neuroinflammation is a leading cause of secondary neuronal injury in neonatal hypoxic-ischemic encephalopathy (HIE). Regulation of neuroinflammation may be beneficial for treatment of HIE and its secondary complications. Gallic acid (GA) has been shown to have anti-inflammatory and antioxidant effects. In this report we found that oxygen-glucose deprivation and/reoxygenation (OGD/R)-induced cell death, and the generation of excessive reactive oxygen species (ROS) and inflammatory cytokines by microglia were inhibited by GA treatment. Furthermore, GA treatment reduced neuroinflammation and neuronal loss, and alleviated motor and cognitive impairments in rats with hypoxic-ischemic brain damage (HIBD). Together, our results reveal that GA is an effective regulator of neuroinflammation and has potential as a pharmaceutical intervention for HIE therapy.

## Introduction

Neonatal hypoxic-ischemic encephalopathy (HIE) is a cerebral hypoxic-ischemic injury caused by perinatal asphyxia with an incidence of approximately 1–8/1,000 (1). Although the incidence of HIE has decreased as improvements have been made in prenatal and neonatal care, HIE remains the leading cause of neonatal death, with survivors later showing severe neurological sequelae such as cerebral palsy, epilepsy, and cognitive impairment (2–6). Treatment of patients with HIE and its sequelae places a heavy burden on families and society, so identifying more effective ways to improve the quality of life of HIE patients and reducing the incidence of sequelae is urgently needed.

Previous studies have shown that HIE induces primary neuronal death and delayed neuronal death. In HIE, delayed neuronal death in the brain is mainly caused by excitotoxicity, oxidative stress, and inflammation (7). As the main immune cells in the brain, microglial cells are activated rapidly after HIE and produce excessive oxidative reactive products such as reactive oxygen species (ROS) and various proinflammatory cytokines, such as interleukin-1β (IL-1β), interleukin-6 (IL-6), and tumor necrosis factor-α (TNF-α), resulting in neuroinflammation and leading to the destruction of the blood-brain barrier and infiltration of peripheral inflammatory cells into the central nervous system, which aggravates neuroinflammation and subsequent neuronal death (8–10). Therefore, controlling the inflammatory response has become a potential approach for the clinical treatment of HIE.

Gallic acid (GA) is a common polyhydroxy phenolic compound in plants. Studies have shown that GA has anti-inflammatory and antioxidant effects and exerts a neuroprotective effect in different models of neurological disease (11–14), but little is known about its effect on HIE. Since neuroinflammation plays an essential role in the occurrence and development of HIE and its sequelae, we hypothesize that GA can alleviate brain damage in HIE by reducing inflammation. In this study, we found that GA could inhibit microglia-mediated neuroinflammation and oxidative stress after OGD/R and reduce neuroinflammation and neuronal loss to improve motor and cognitive abilities in a hypoxic-ischemic brain damage (HIBD) model.

## Materials and methods

### Cell culture

BV2 cells were cultured in DMEM containing 10% FBS (Excell Bio, FSP500) and placed in a 37 °C incubator with 5% CO2. GA (MCE, HY-N0523) was dissolved in DMSO to generate a 100 mg/ml stock solution and diluted with PBS to 100 mM before use. To establish a model of oxygen glucose deprivation/reoxygenation (OGD/R), BV2 cells were cultured in a hypoxic incubator and treated with glucose-free DMEM (Gibco, A1443001) with or without GA for 4 h. Afterward, the medium was replaced with normal medium with or without GA, and the cells were placed in a normal incubator for 1 h to simulate the reperfusion process. Control cells were cultured for the same duration under normal conditions.

### CCK8 assay

Cell viability was measured by a Cell Counting Kit-8 (MCE, HY-K0301) according to the manufacturer's instructions. BV2 cells were seeded into 96-well plates at an appropriate density, and after treatment, 10 µl of CCK8 solution was added to each well. The absorbance at 450 nm was measured with a microplate reader after incubation for 1 h.

### Flow cytometry

ROS levels were measured by flow cytometry. Briefly, cells were collected, incubated with 10 µM DCFH-DA (Beyotime, S0033S) for 20 min, washed with PBS 3 times and then analyzed by flow cytometry.

### Real-time quantitative PCR (qPCR)

RNA was extracted using a BioTeke kit (BioTeke, RP1202), and the RNA was then reverse-transcribed into cDNA (Takara, RR047A). TB Green Premix Ex Taq (Takara, RR820A) and gene-specific primers were used to amplify the cDNA. The following primers for mouse were used: *β*-actin (forward, 5′-ACTGTCGAGTCGCGTCC and reverse 5′-CTGACCCATTCCCACCATCA); IL-1β (forward, 5′-TGCCACCTTTTGACAGTGATG and reverse 5′-ATGTGCTGCTGCGAGATTTG); IL-6 (forward, 5′-GAGCCCACCAAGAACGATAG and reverse 5′-GTTGTCACCAGCATCAGTCC). The following primers for rat were used: *β*-actin (forward, 5′-GTCCACCCGCGAGTACAACCTTCT and reverse 5′-TCCTTCTGACCCATACCCACCATC); IL-1β (forward, 5′- TGAGGCTGACAGACCCCAAAAGAT and reverse 5′-GCTCCACGGGCAAGACATAGGTAG); IL-6 (forward, 5′-AGCCACTGCCTTCCCTACTTCA and reverse 5′-GCCATTGCACAACTCTTTTCTCA). The data were processed by the 2^−*ΔΔ*CT^ method.

### HIBD animal model and treatment

All animal studies were conducted in accordance with the Guide for the Care and Use of Laboratory Animals of the Ethics Committee of Chongqing Medical University. Animals were blindly grouped and analyzed. The experimental procedures were approved by the Animal Study Committee of the Children's Hospital of Chongqing Medical University. Seven-day-old Sprague-Dawley (SD) rats were used to construct the HIBD model, a well-validated animal model (15), as described previously (16). Rats were anesthetized by intraperitoneal injection of sodium pentobarbital at a dose of 30 mg/kg. The left carotid artery of the neonatal rats was ligated, and the rats were then placed in a hypoxic cage (8% O2 + 92% N2) at 37 °C for 2.5 h after resting for approximately 1 h. The rats in the sham group underwent left carotid artery isolation without ligation and hypoxia. After hypoxia exposure, the pups were returned to their home cages and care for by their mothers until day P21. All the rats had free access to water and food and were kept in a constant-temperature room on a 12 h light/12 h dark cycle.

**Figure 1:**
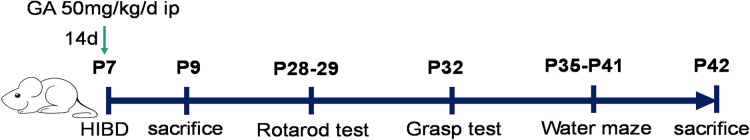
The timeline of HIBD animal experimental procedure.

The animals were randomly divided into four groups: the sham + saline, sham + GA, HIBD + saline, and HIBD + GA groups. GA was dissolved in saline to a concentration of 10 mg/mL and injected intraperitoneally at a dose of 50 mg/kg for 14 days. The first injection was performed immediately after construction of the HIBD model (Figure 1).

### Rotarod test

Three weeks after HIBD model construction, the rotarod test (16) was performed (Figure 1). Two adaptative training sessions were performed first, and the rats that did not cooperate were excluded. Then, ten consecutive rotarod trials were performed the day after adaptive training. The rotarod was accelerated from 5 r/min to 60 r/min over 3 min, and the time that each rat stayed on the rotarod was recorded. The instrument was cleaned with 75% alcohol after each test.

### Grasping test

Two days after the rotarod test, the rats were subjected to the grasping test (16) to evaluate muscle strength (Figure 1). The left or right forelimb of each rat was placed on the grasp meter. Then, the rats were pulled back until they could no longer grasp the meter, and the maximum tension was recorded. The measurement was repeated 10 times for each rat's unilateral forelimb.

### Morris water maze (MWM) test

The MWM test (17) was performed after the grasping test to assess the spatial learning and memory of the rats (Figure 1). On the first day, the rats underwent adaptative training, during which they were placed in the water maze and allowed to swim for 60 s. A spatial learning trial was performed on each of the subsequent 5 days; the rats were placed in the water facing the pool wall at staggered entry points (1–3–2–4/4–2–3–1). The time required for the rats to find the platform was recorded. If a rat did not find the platform within 60 s, it was guided to the platform and allowed to stay on it for 10 s. On the last day, a probe test was performed. The hidden platform was removed, and the rats were placed in the water in the quadrant opposite the platform quadrant. The time the rats spent in the platform quadrant and the number of times they crossed the platform location were recorded. All the data were recorded by an Any-maze tracking system.

### Immunofluorescence

After the behavioral tests, the rats were anesthetized with urethane, and the left hemisphere of the brain was fixed with 4% paraformaldehyde for several days after transcardial perfusion with 0.9% saline. Thereafter, the brain tissues were dehydrated with 20% and 30% sucrose until it sank to the bottom of the tube. Next, the brain tissues were sectioned into 20 μm slices. Six to nine slices per brain were washed in PBS, treated with blocking solution (Beyotime, P0260) for approximately 30 min and then incubated overnight at 4 °C with anti-NeuN primary antibodies (1:400, Abcam, ab177487). The slices were subsequently incubated with secondary antibodies (1:500, Invitrogen, A21206) for 90 min the next day. Images were taken with an Olympus full-slide scanner, and the number of neurons in the CA1 region was counted with ImageJ.

### Statistical analysis

SPSS 19.0 software was used to analyze the data. The data in this study were all quantitative and expressed as the means ± standard errors (SEMs). Student's t test was used for comparisons between two groups, while one-way ANOVA followed by the LSD test was used for comparisons among multiple groups. Differences in data from the spatial learning trials of the MWM test were assessed by two-way repeated-measures ANOVA with matched subjects followed by the LSD multiple comparison *post hoc* test. *P *< 0.05 was considered statistically significant.

## Results

### GA increased the viability of Bv2 cells after OGD/R

**Figure 2:**
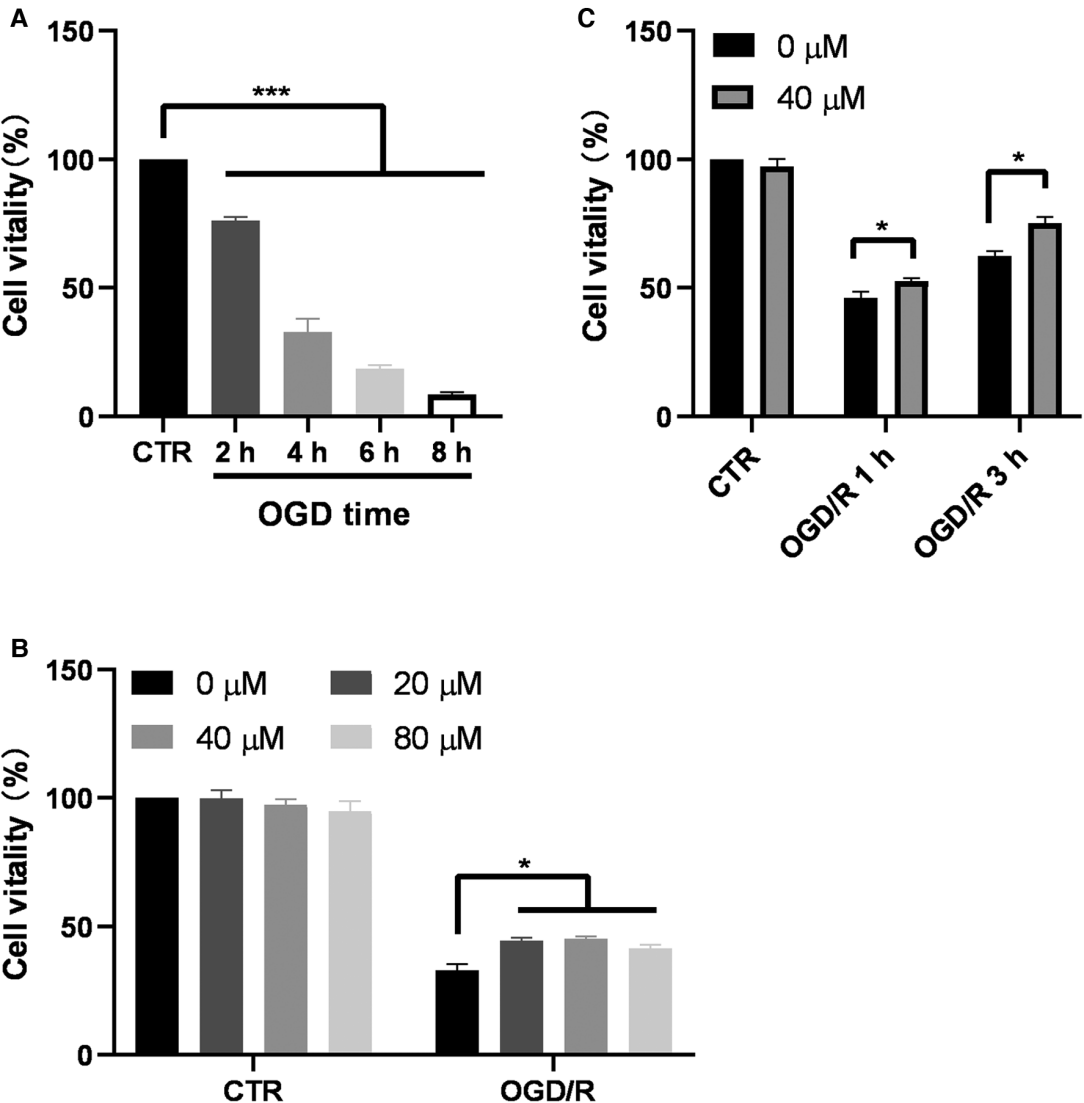
**Ga increases BV2 cell viability after OGD/R. (A)** BV2 cells were exposed to OGD for 2, 4, 6 and 8 h and the cell viability was detected by CCK-8 assay (*n* = 6). **(B**) Effect of different GA concentrations on cell viability of control cells for 7 h (CTR) and the cells treated with OGD for 4 h followed by reoxygenation for another 3 h (OGD/R). GA treatment significantly increased the survival rate of OGD cells without affecting Normal cells (*n* = 4). (**C)** GA treatment improved the cell viability after OGD for 4 h and reoxygenation for 1 and 3 h (*n* = 4). CTR, control. Data are presented as mean ± SEM. *P < 0.05, **P < 0.01, ***P < 0.001.

To investigate the effect of GA on cell viability, BV2 cells were first subjected to OGD, and then CCK8 assay was performed. The results of the CCK8 assay showed that after 2, 4, 6, and 8 h of OGD, cell viability significantly decreased to 76.19% ± 1.38%, 32.95% ± 5.09%, 18.47% ± 1.43%, and 8.43% ± 2.56%, respectively (Figure 2A). OGD for 4 h was chosen as the optimal condition for GA treatment. In the absence of OGD/R, GA treatment had no effect on cell viability (Figure 2B). However, after 3 h of reoxygenation, treatment with 20, 40 or 80 µM GA markedly increased cell viability to 44.51% ± 2.22%, 45.18% ± 2%, and 41.51% ± 2.76%, respectively from 33% ± 4.96% (Figure 2B). To examine the effect of GA on cell viability after reoxygenation for different durations, cells were treated with 40 µM GA. The cell viability was decreased to 46.18% ± 4.7%, and 62.4% ± 3.93% after 1 h and 3 h of reoxygenation, respectively, and GA significantly increased the survival rate of OGD-treated cells to 52.7% ± 2.27% and 75.04% ± 5.01% after 1 h and 3 h of reoxygenation, respectively (Figure 2C). These data indicated that GA protected BV2 cells from OGD/R.

### GA inhibited OGD/R-induced ROS and proinflammatory cytokine production in Bv2 cells

**Figure 3:**
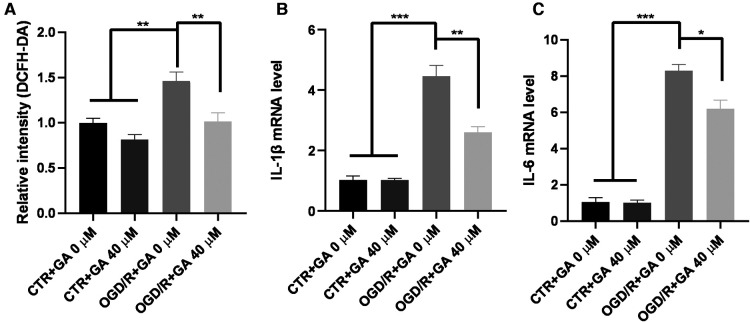
**Ga inhibits OGD/R-induced ROS and inflammatory cytokines generation. (A)** Quantification of ROS generation in BV2 cells. The mean fluorescence intensity of the control group without GA treatment was used to normalize for analysis. GA treatment reduced the overproduction of ROS induced by OGD/R (*n* = 5). The increased mRNA levels of IL-1β **(B)** and IL-6 **(C**) in OGD/R group were rescued with GA treatment (*n* = 3). Data are presented as mean ± SEM. *P  < 0.05, **P < 0.01, ***P < 0.001.

Considering the influence of microglial activation in the acute phase on the progression of the disease, OGD for 4 h and reperfusion for 1 h were chosen as the conditions for further study. To determine whether the protective effect of GA is related to the decreases in ROS and proinflammatory cytokine levels, the levels of ROS, IL-1β, and IL-6 in BV2 cells were measured (Figure 3). The levels of ROS, IL-1β and IL-6 mRNA significantly increased in the OGD/R group, while treatment with 40 µM GA significantly decreased their levels.

### GA decreased neuroinflammation in HIBD model rats

**Figure 4:**
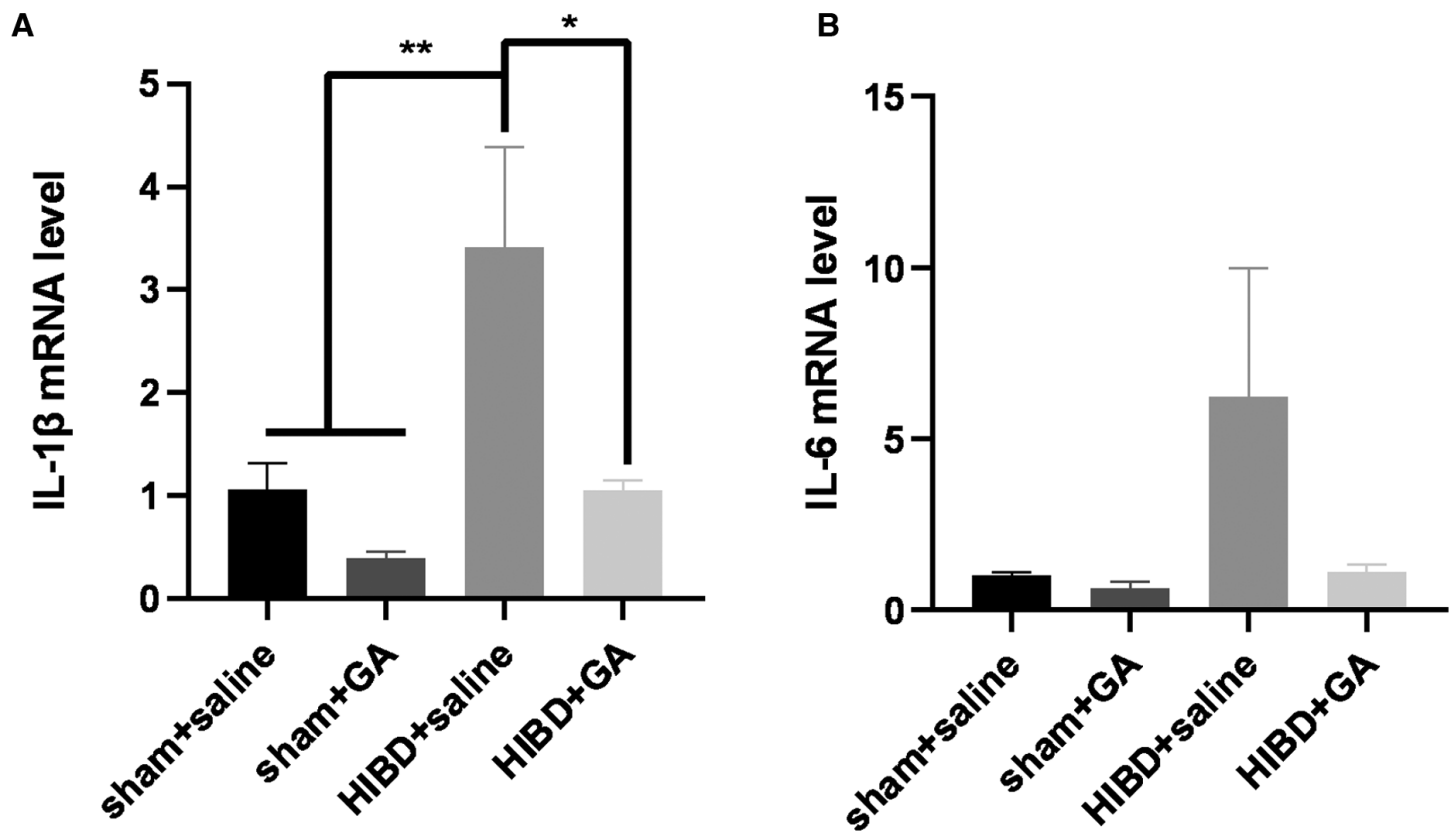
**Ga decreases the neuroinflammation in HIBD rats.** The rats were sacrificed 48 h after modeling, and RNA was extracted to detect the expression of proinflammatory cytokines. The increased mRNA levels of IL-1β **(A)** and IL-6 **(B)** in HIBD rats were rescued with GA treatment (*n* = 3). Data are presented as mean ± SEM. *P < 0.05.

The *in vitro* results demonstrated that GA could exert a neuroprotective role by inhibiting OGD/R-induced ROS and proinflammatory cytokine production in BV2 cells. To determine whether GA could inhibit neonatal hypoxic-brain injury by exerting an anti-inflammatory effect, the levels of proinflammatory cytokines were also measured *in vivo*. HIBD model was established in 7-day-old SD rats, and RNA was extracted from the brains of rats in the different groups 48 h after modeling. The HIBD model rats exhibited higher expression of IL-1β and IL-6 (3.41 ± 0.97 and 6.25 ± 3.75 fold for IL-1β and IL-6, respectively) while the expression of IL-1β and IL-6 was decreased in response to GA treatment (1.06 ± 0.96 and 1.11 ± 0.23 fold for IL-1β and IL-6, respectively) (Figure 4).

### GA reduced neuronal loss in HIBD model rats

**Figure 5:**
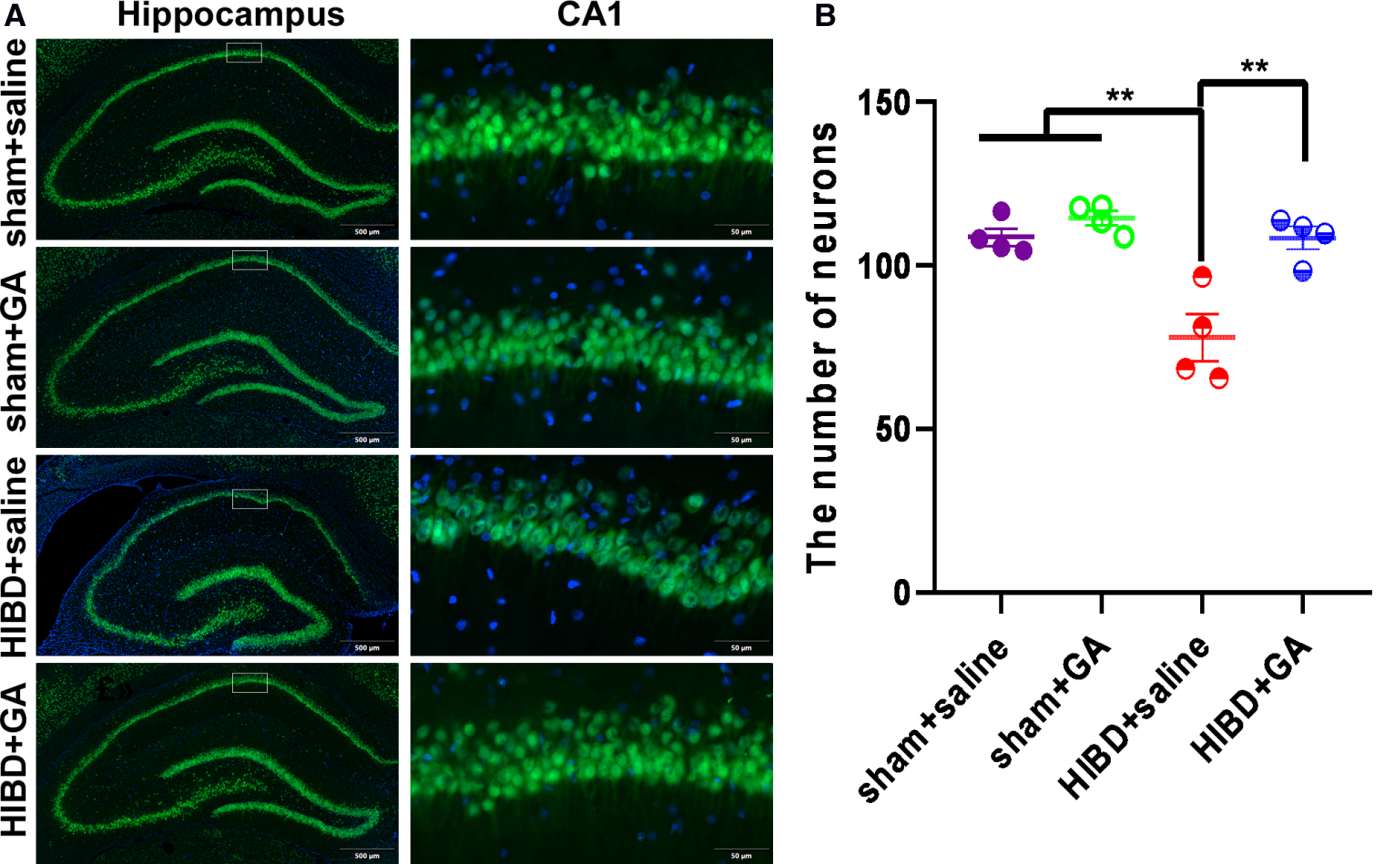
**Ga reduces neuronal loss in CA1 region of HIBD rats.** The HIBD model rats were sacrificed and the brain tissues were sectioned. The neurons were visualized by immunofluorescence staining with the neuron marker NeuN and the number of neurons in the hippocampal CA1 region were counted. **(A**) Representative immunofluorescence images of different groups. Scale bar: 500 *μ*m for the left image and 50 *μ*m for the right image (*n* = 4). **(B**) Quantification of NeuN positive cells. The number of neurons were significantly decreased in saline-treated HIBD rats compared to the rats in sham groups, which could be saved by GA treatment (*n* = 4). Data are presented as mean ± SEM. **P < 0.01.

Five weeks after HIBD rats were subjected to behavioral tests, brain tissue was sectioned for immunofluorescence staining with the neuronal marker NeuN to determine the number of neurons in the hippocampal CA1 region. The number of neurons in the CA1 region was significantly reduced in the HIBD group compared with the sham group, but was dramatically increased in response to GA treatment (108.63 ± 2.73, 114.49 ± 2.16, 77.93 ± 7.09, 108.42 ± 3.46 for sham + saline, sham + GA, HIBD + saline, and HIBD + GA, respectively) (Figure 5). The results suggested that GA protected neurons from HIBD.

### GA ameliorated HIBD-induced motor dysfunction and muscle strength reduction

**Figure 6:**
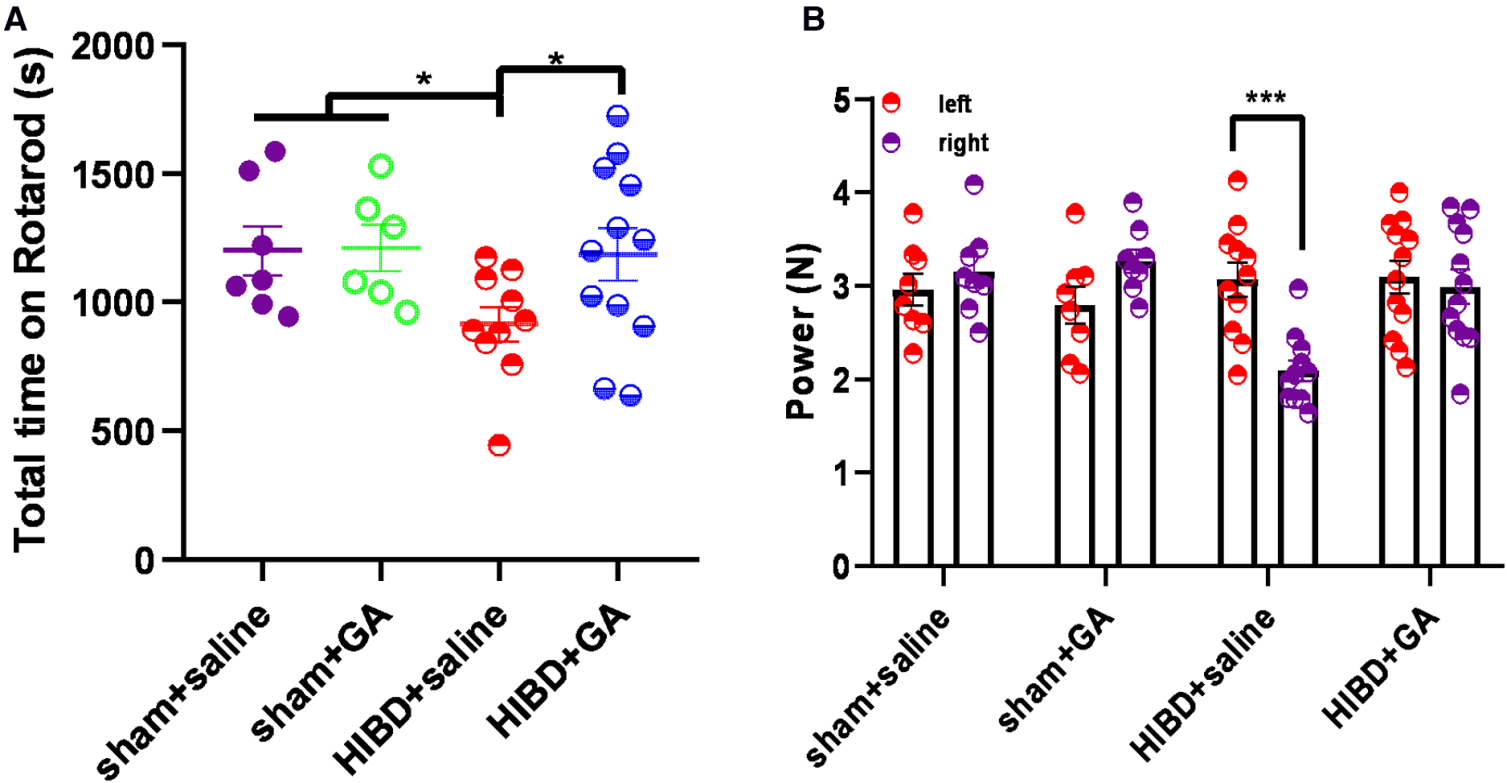
**Ga alleviates the deficits of motor ability and muscle strength in HIBD model rats. (A**) Rats were subjected to HIBD at the postnatal 7th day, GA or saline was injected daily for 2 weeks and the rotarod test was conducted at the 28th day. After the first day of adaptive training, the rats were put on the rotarod for 10 trails on the following day and the time on the rotarod was recorded. GA-treated HIBD rats increased the total time spent on the rotarod compared with the saline-treated HIBD rats (sham + saline, *n* = 7; sham + GA, *n* = 6; HIBD + saline, *n* = 10; HIBD + GA, *n* = 12). **(B**) The muscle strength of the left and right fore-limb was evaluated by grasping test. The imbalance muscle strength of the upper limbs caused by HIBD was saved by GA treatment (sham + saline, *n* = 8; sham + GA, *n* = 8; HIBD + saline, *n* = 11; HIBD + GA, *n* = 12). Data are presented as mean ± SEM. *P < 0.05, **P < 0.01, ***P < 0.001.

To test the protective effects of GA, the motor ability and muscle strength of the rats were measured by the rotarod test and grasping test. In the rotarod test, the total time that the rats in the HIBD + saline group stayed on the rotarod was significantly reduced, which was rescued by GA treatment (1200.46 ± 95.81, 1210.77 ± 89.57, 914.19 ± 67.06, and 1185.31 ± 101.44 s for sham + saline, sham + GA, HIBD + saline, HIBD + GA, respectively) (Figure 6A). The data indicated that GA treatment did not affect the motor ability of the rats in the sham group but significantly ameliorated HIBD-induced motor impairment.

In the grasping test, the muscle strength of the right fore-limb (2.09 ± 0.11 N) was significantly decreased compared with that of the left forelimb (3.07 ± 0.18 N) in the HIBD + saline group, but no difference was observed after GA treatment (3.1 ± 0.18 N for left fore-limb and 2.99 ± 0.18 N for right fore-limb). In the sham groups, regardless of whether GA was administered, there was no difference in muscle strength between the left and right sides (2.96 ± 0.17 and 3.15 ± 0.17 N, 2.79 ± 0.2 and 3.27 ± 0.12 N for sham + saline and sham + GA, respectively) (Figure 6B).

### GA alleviated HIBD-induced cognitive impairment

**Figure 7:**
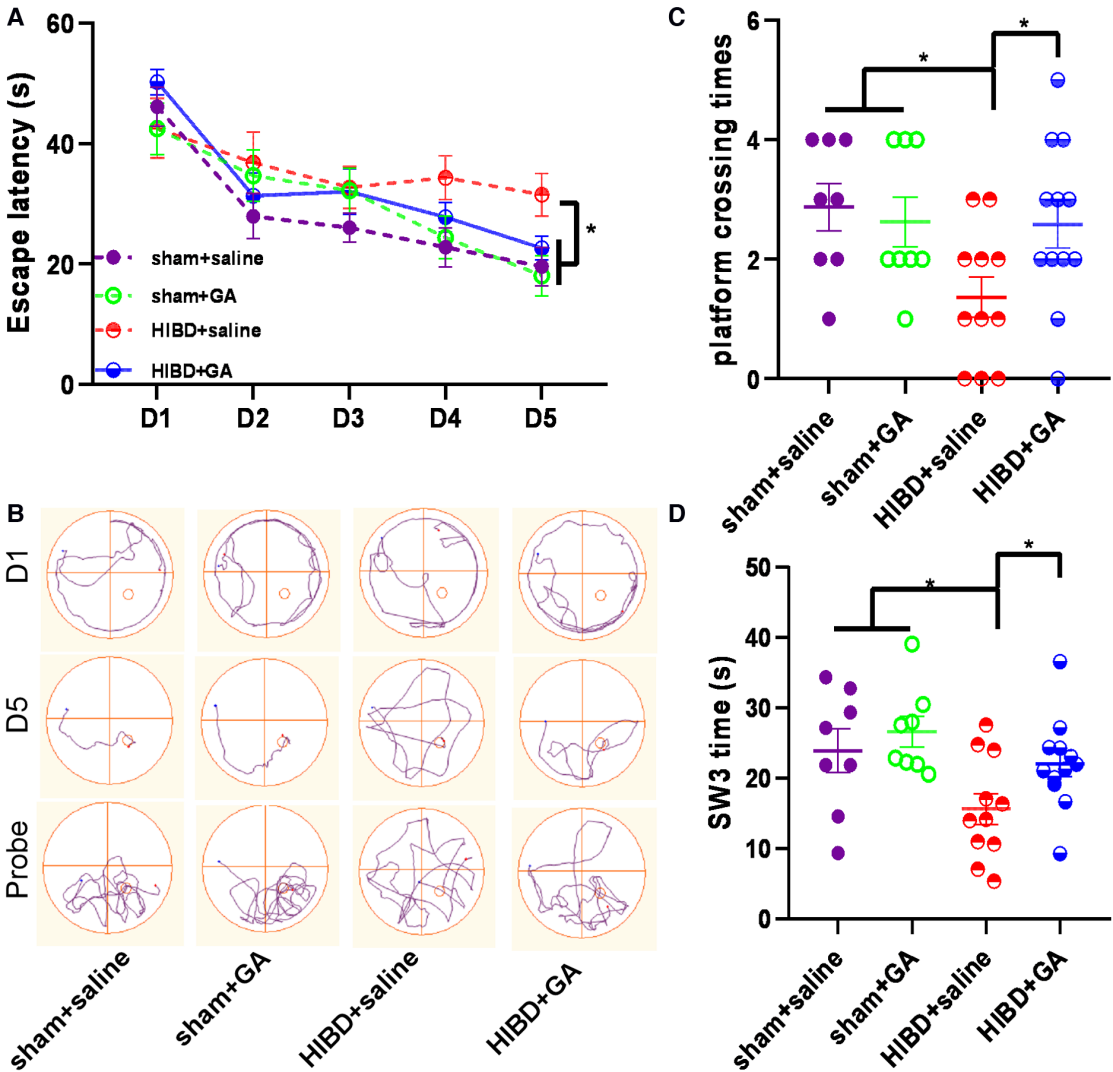
**Ga improves the learning and memory of HIBD rats.** MWM test consists of 1 day of adaptive trials and 5 days of hidden platform trials, plus a probe trial 24 h after the last hidden platform trial. Animal movement was tracked and recorded by ANY-maze tracking software (sham + saline, *n* = 8; sham + GA, *n* = 8; HIBD + saline, *n* = 11; HIBD + GA, *n* = 12). **(A**) In hidden platform tests, rats were trained with 4 trials per day for 5 days. GA-treated HIBD rats showed a shorter latency to escape onto the hidden platform on fifth days compared with the saline-treated HIBD rats. **(B**) Tracks of the rat movement in MWM test on different days. In the probe trial, the number of crossing platforms **(C**) and the time spent in the platform quadrant **(D)** of GA-treated HIBD rats were significantly more than the rats in saline-treated HIBD group. Data are presented as mean ± SEM. *P < 0.05.

HIBD causes learning and memory disorders in animals (18–21). To investigate whether GA could improve cognitive functions, the spatial learning and memory of rats were evaluated by the MWM test. As shown in Figure 7A, the escape latency of rats in the HIBD + saline group was longer than that of rats in the other three groups, and the escape latency on day 5 was somewhat decreased by GA treatment (19.57 ± 3.18, 18.08 ± 3.35, 31.52 ± 3.54, and 22.61 ± 1.95 s for sham + saline, sham + GA, HIBD + saline, HIBD + GA, respectively). GA treatment also significantly increased the time spent in the platform quadrant (23.95 ± 3.09, 26.63 ± 2.17, 15.66 ± 2.19, and 22.11 ± 1.86 s for sham + saline, sham + GA, HIBD + saline, and HIBD + GA, respectively) (Figure 7D), and the number of platform crossings (2.88 ± 0.4, 2.63 ± 0.42, 1.36 ± 0.34, and 2.58 ± 0.4 times for sham + saline, sham + GA, HIBD + saline, and HIBD + GA, respectively) following HIBD. (Figure 7C). These data suggested that GA treatment alleviated HIBD-induced cognitive impairment.

## Discussion

GA, a plant-derived phenolic acid with anti-inflammatory and antioxidant effects, is highly safe and has few side effects (22, 23). GA has been reported to exert a protective effect in many diseases, such as ischemic hypoxic injury in adult rats (24) or mice (25), Parkinson's disease (11), diabetes (26), and tumor (27), but little is known about its protective effect in newborn mice. HIE is not a single event, but a sustainable process. Inflammation has been considered as an important contributor in the pathophysiology of cerebral hypoxic ischemia (HI) injury (7, 28, 29). Previous studies have shown that the increased levels of inflammatory cytokines in the cerebrospinal fluid are associated with adverse neurological outcomes in children with HIE (30, 31). Neuroinflammation caused by HIE further leads to cell death and ultimately dysfunction (7, 32). Various drugs targeting neuroinflammation have been tested in animal models of perinatal brain damage. Several compounds that are already used in clinical such as melatonin and COX inhibitors have shown promising neuroprotective properties by inhibiting neuroinflammation (28). So timely inhibition of the inflammatory response in the early stage of HIE is critical to reduce injury.

Microglial cells are key immune cells that are involved in maintaining brain homeostasis (33). Previous studies have shown that microglia play a dual role in neuronal injury and recovery, which is associated with their phenotype; specifically, M1 microglia promote the release of inflammatory cytokines and aggravate injury, while M2 microglia can release anti-inflammatory cytokines to play a protective role (34, 35). HI promotes the polarization of microglia to the M1 phenotype and the release of proinflammatory factors such as TNF-α, IL-1β, and IL-6, which have toxic effects on nerve cells in the surrounding area (36, 37). In addition, excess production of ROS by activated microglia induces oxidative damage in the developing brain (38). Our study showed that OGD/R activated microglia and promoted the release of inflammatory cytokines and ROS, whereas GA treatment alleviated the damage induced by OGD/R; moreover, GA itself did not affect the inflammatory response and oxidative stress in normal BV2 cells, suggesting that GA could play a neuroprotective role by reducing neuroinflammation in OGD/R-treated BV2 cells.

Neuroinflammation caused by HI results in neuronal death, and neuronal death results in motor and cognitive impairments in both animal models and humans (39–41). Interestingly, in this study we found that GA could reduce neuroinflammation, neuronal loss and neurological dysfunction caused by HI. In addition to exerting positive effects in the HIBD model, GA has been reported to improve the cognitive function of patients with Alzheimer's disease and reduce motor dysfunction caused by lead and aflatoxin B1 exposure (13, 42–44). These evidences confirmed the neuroprotective effect of GA in HIBD.

GA was found to downregulate HI-induced proinflammatory genes *in vitro* and *in vivo*. Considering that M1 microglia can promote the release of inflammatory cytokines and aggravate injury, GA might play a protective role by affecting the polarization of microglia, thus decreasing the levels of inflammation-related genes and ROS. However, the specific mechanism through which GA regulates neuroinflammation after HI needs further in-depth study.

## Conclusion

The evidence provided in this study indicates that, by inhibiting abnormal inflammatory activation, GA reduces HIBD-induced neuronal death and improves the motor and cognitive impairments following a hypoxic-ischemic insult. The data from our investigation support that GA is neuroprotective and may be a potentially effective drug for treatment of HIE and prevent its sequelae.

## Data Availability

The original contributions presented in the study are included in the article, further inquiries can be directed to the corresponding author/s.
